# Wafer scale BN on sapphire substrates for improved graphene transport

**DOI:** 10.1038/s41598-018-27237-z

**Published:** 2018-06-11

**Authors:** Shivashankar Vangala, Gene Siegel, Timothy Prusnick, Michael Snure

**Affiliations:** 1 Air Force Research Laboratory, Sensors Directorate, Wright Patterson AFB, 45433 USA; 2 KBR Wyle Laboratories, Beavercreek, OH 45433 USA

## Abstract

Wafer scale (2”) BN grown by metal organic chemical vapor deposition (MOCVD) on sapphire was examined as a weakly interacting dielectric substrate for graphene, demonstrating improved transport properties over conventional sapphire and SiO_2_/Si substrates. Chemical vapor deposition grown graphene was transferred to BN/sapphire substrates for evaluation of more than 30 samples using Raman and Hall effects measurements. A more than 2x increase in Hall mobility and 10x reduction in sheet carrier density was measured for graphene on BN/sapphire compared to sapphire substrates. Through control of the MOCVD process, BN films with roughness ranging from <0.1 nm to >1 nm were grown and used to study the effects of substrate roughness on graphene transport. Arrays of graphene field effect transistors were fabricated on 2” BN/sapphire substrates demonstrating scalability and device performance enhancement.

## Introduction

Due to its amazing properties, graphene continues to be of great interest more than a decade after its experimental discovery^1^. Unfortunately, interactions between graphene and its environment, including the substrate, greatly degrade its transport properties. Substrate induced scattering from charged impurities, roughness, and phonons will reduce mobility^2,3^. The most successful approach to mitigate these effects has been the use of a thin van der Walls (vdW) buffer layer, like hBN^4,5^ or MoS_2_^6^, preserving the mobility of the graphene to values near those of suspended material. Of the available vdW materials, hBN is one of the only insulators and due to is dielectric properties (comparable to SiO_2_^7^), high surface optical phonon energy (169 eV)^8^ and atomically smooth surface; it has been the most successful and widely used material for preserving and protecting the intrinsic properties of 2D materials (graphene, phosphorene, MoS_2_, …)^4–6,9–14^. The overwhelming majority of this work has used hBN flakes exfoliated from high quality bulk materials. The high purity and crystal quality of this exfoliated material has given rise to amazing properties; however, due to the small size, low yield and poor reproducibility of these exfoliated flakes this process is not scalable or suitable for device fabrication beyond the laboratory.

The need for large area high quality hBN thin films for 2D substrates, dielectrics, and tunneling barriers has generated considerable upswing in hBN growth. High quality hBN thin films from mono to many layers thick over large areas have been produced using chemical vapor deposition (CVD) using both metallic^15–18^ and insulating substrates^19–22^. Taking advantage of the catalytic nature of metal substrates, like Cu and Ni, high quality material can be grown at quite low temperatures (<1000 °C). Since this process it typically surface catalyzed the controlled growth of mono-to few-layer hBN is possible^23^. The hBN grown on metal films can then be transferred to insulating substrates for further processing and characterization. Improved transport in graphene has been demonstrated using such transferred hBN films^24,25^ as compared to conventional substrates. Lee *et al*.^24^ demonstrated a 3x increase in graphene mobility when a few layer hBN buffer was inserted on SiO_2_/Si. Back gated graphene field effect transistors (FET) on hBN, grown on Fe substrates showed improved mobility and reduced intrinsic doping as compared to similar devices directly on SiO_2_/Si^25^. For many of the applications described above hBN grown on a metal will inevitably require transfer to insulating substrates adding complexity, sources of contamination, and damage from the fabrication process.

Alternatively, hBN films grown directly on insulating substrates do not require transfer prior to device processing. Due to the challenges of growing hBN directly on an insulating substrate, such as sapphire, it has been much less explored than its counterpart grown on metals. Growing on sapphire it is much more challenging to produce continuous ultra-thin films of mono– or bi-layer thickness, and as such, most reports demonstrate films from few to tens of nanometers thick^20,26,27^. The formation of large hexagonal wrinkles has also been observed in BN grown on sapphire due to the thermal expansion mismatch between BN and the substrate^20,28^. These features make BN films grown on sapphire distinct from those on metals. Jang *et al*. reported growth of smooth few-layer hBN on sapphire and demonstrated a 2x increase in graphene field effect mobility as compared to graphene on SiO_2_/Si^29^. Now with the availability of high quality large area CVD grown BN films on sapphire, more work is needed to explore their applications as substrates and dielectrics for 2D electronics.

In this paper, we investigate the use of few-layer sp^2^-BN on sapphire as a weakly interacting substrate for graphene. The basal plane of sp^2^ bonded BN forms the same honeycomb lattice as graphene and can form atomically thin and smooth layers. In multilayer BN, layers are known to stack hexagonal (AB stacking), rhombohedral (ABC stacking), or turbostratic (random stacking) with very small differences in inter planar spacing. For this work, CVD grown graphene was transferred to wafer scale sp^2^ BN/sapphire (now referred to as BN) grown by metal organic chemical vapor deposition (MOCVD) to study the effects on transport properties by Hall and FET device measurements. The effect of BN roughness was also investigated using BN films with roughness from <0.1 nm to >1 nm. Results show a more than 2x increase in graphene mobility when transferred to BN on sapphire substrate with a strong dependence on substrate induced doping related to BN roughness.

## Results

**Figure 1:**
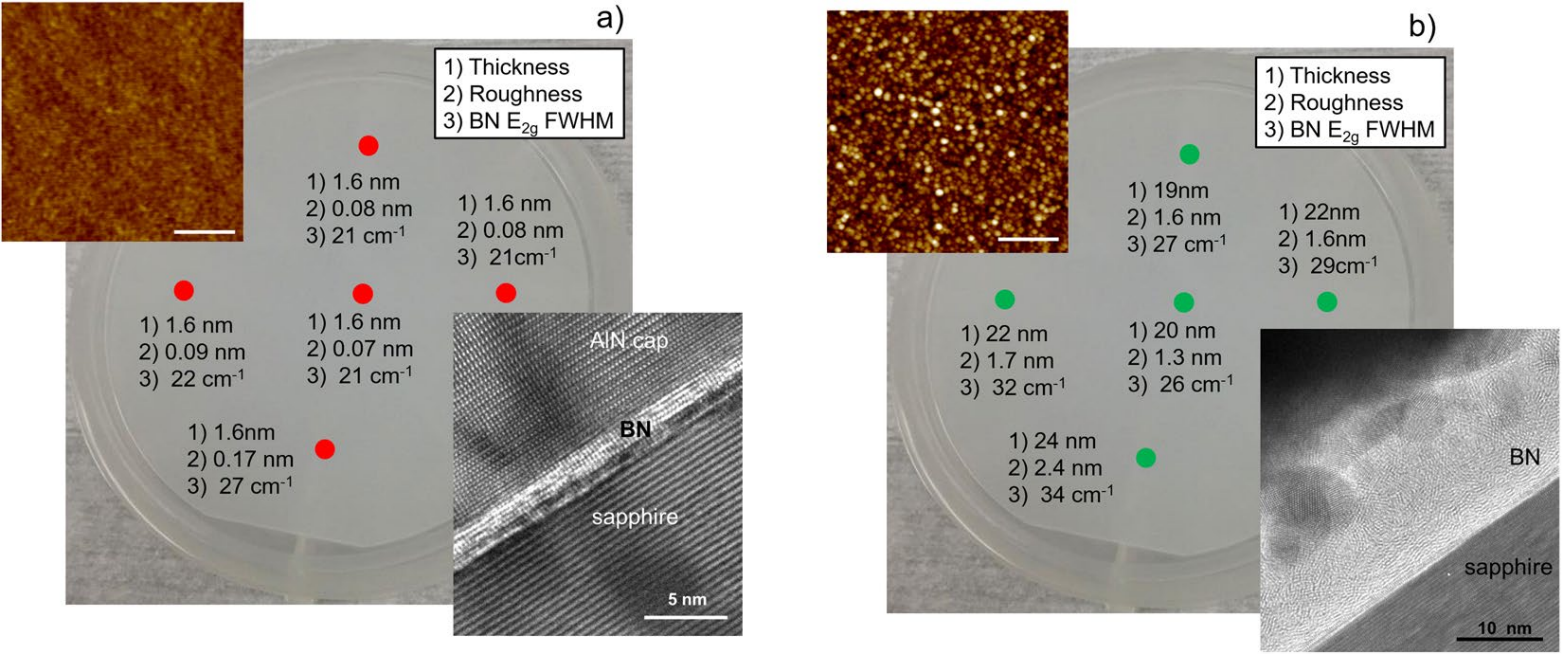
Boron Nitride on sapphire as a substrate for graphene. Optical image with BN thickness, roughness, and full width at half maximum of sp^2^ BN E_2g_ Raman mode at five points for (**a**) smooth and (**b**) rough BN/sapphire substrates. Top left insets are AFM images for the center of each BN/sapphire substrate (scale bar 250 nm) and bottom right are cross-sectional TEM images.

For application as a graphene substrate, BN films need to be atomically smooth, atomically flat, insulating and uniform over large areas. Growth of BN on sapphire by MOCVD at low pressure (20 Torr) and high V/III ratios (>2250) has been shown to produce films fitting these parameters with excellent thickness uniformity (1.6 + /−0.1 nm) and low roughness (<0.1 nm) over large areas (Fig. 1a). Under these low-pressure high V/III ratio conditions growth has been shown to be self-terminating (Supplementary Fig. S1) resulting in c-oriented films, which are consistently 4 mono-layers thick^26^. Reducing the V/III ratio or increasing the pressure disrupts these self-terminating conditions resulting in thicker, rougher (>1 nm) films with a random orientation (Fig. 1b)^30^. Varying the V/III ratio across a range of 450 to 2250 produces films with RMS roughness from a few nanometer to atomically smooth (Supplementary Fig. S2). From TEM the BN interlayer spacing was measured to be~ 3.45 and 3.5 Å for the smooth (high V/III ratio) and rough (low V/III ratio) BN films, respectively, which is larger than that for hBN (3.35 Å) indicating disorder in the stacking sequence. Raman spectroscopy of these films (Supplementary Fig. S3) shows the characteristic E_2g_ mode of sp^2^-bonded BN at 1369 cm^−1^ ^30^ and X-ray photoelectron spectroscopy (Supplementary Fig. S4) shows B 1 s and N 1 s peaks at a binding energy of 190.2 and 397.6 eV consistent with B-N bonding^16^ (see ref.^30^ for more BN material details). From XPS analysis, the B/N was measured to be 1/1 to 1.05 across a range of samples. To demonstrate uniformity across each wafer Fig. 1a,b gives the measured roughness, thickness and full width at half maximum (FWHM) from the sp^2^-BN E_2g_ mode at 5 points across 2” BN/sapphires wafers. The atomically smooth BN/sapphire is well suited for application as a substrate for 2D materials, while rougher films are useful to study the effects of substrate roughness on graphene transport.

**Figure 2:**
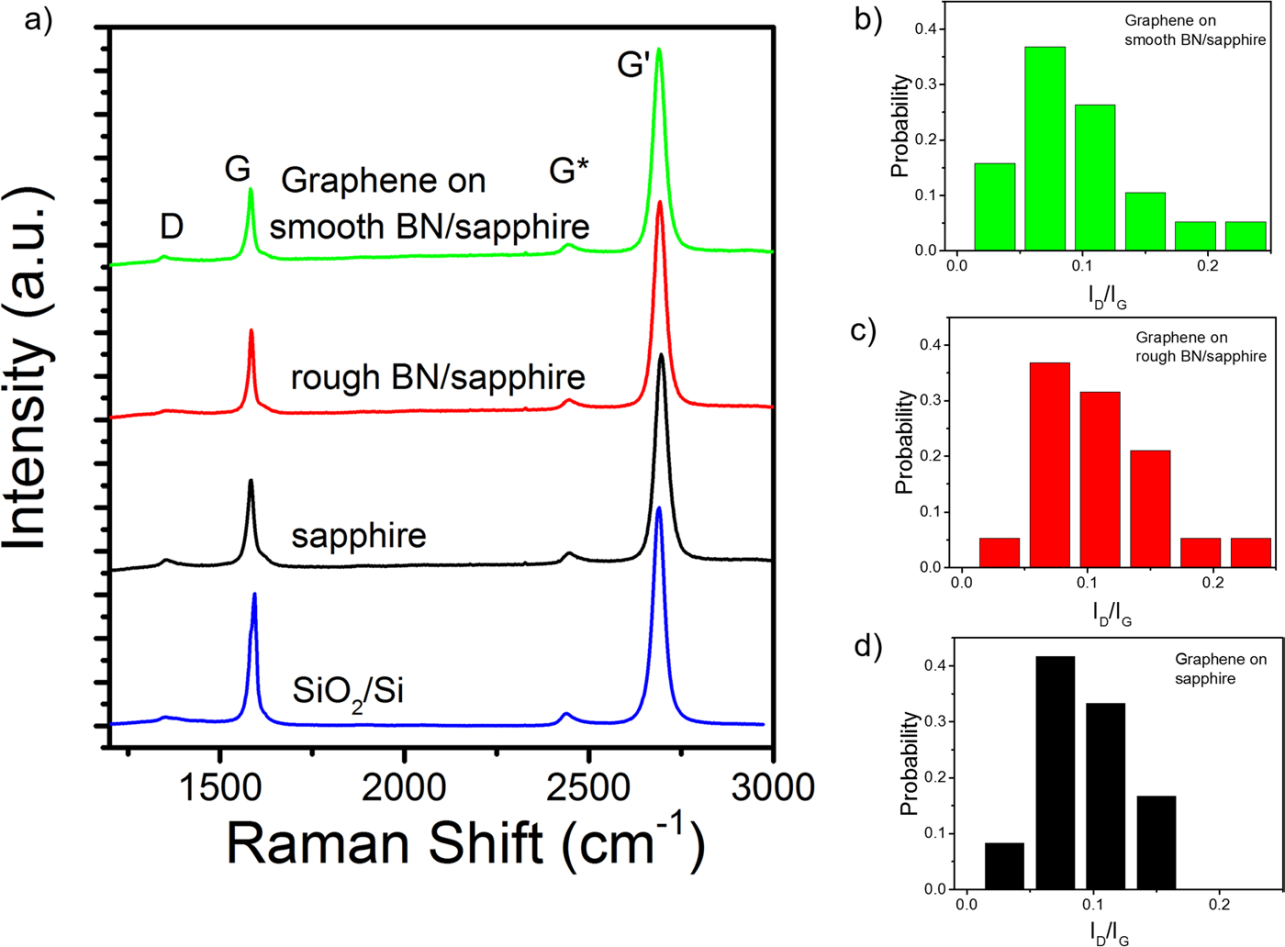
Raman analysis of graphene on various dielectric substrates. (**a**) Representative Raman spectra from graphene on SiO_2_/Si, sapphire, rough BN/sapphire and smooth BN/sapphire substrates. Measured I_D_/I_G_ distribution from graphene films on (**b**) 11 smooth BN/sapphire, (**c**) 9 rough BN/sapphire, and (**d**) 6 sapphire substrates.

Monolayer graphene films grown by CVD on Cu were transferred to 20 different BN on sapphire substrates with BN roughness ranging from >1 nm to <0.1 nm to study the effects of BN roughness and morphology on graphene. Half of the BN/sapphire substrates were grown under self-terminating conditions and have measured roughness <0.1 nm. Graphene was also transferred to sapphire and SiO_2_/Si as reference substrates. Raman spectroscopy of graphene films was used to compare the different dielectric substrates. Figure 2a shows typical graphene spectra taken from films on each substrate. Analysis of these graphene samples shows the substrate has limited effect on G and G’ peak position (Supplementary Fig. S5). Characteristic of all samples is a small defect peak and a G’/G intensity ratio of ~2 indicating films are of good structural quality and predominately monolayer. Raman was also used to evaluate variation between samples on similar substrates as a method to qualify reproducibility of the BN MOCVD growth and graphene transfer processes. The D/G intensity ratio (I_D_/I_G_) taken from multiple spots on each sample was used for comparison, Fig. 2b–d. On sapphire the mean I_D_/I_G_ is 0.09 with a standard deviation (STD) of 0.05 across 6 samples. Comparable values of I_D_/I_G_ of 0.1 with a STD of 0.05 were found for the samples transferred onto both the smooth and rough BN/Sapphire substrates indicating that the graphene is both uniform and undamaged during the transfer process regardless of the substrate.

**Figure 3:**
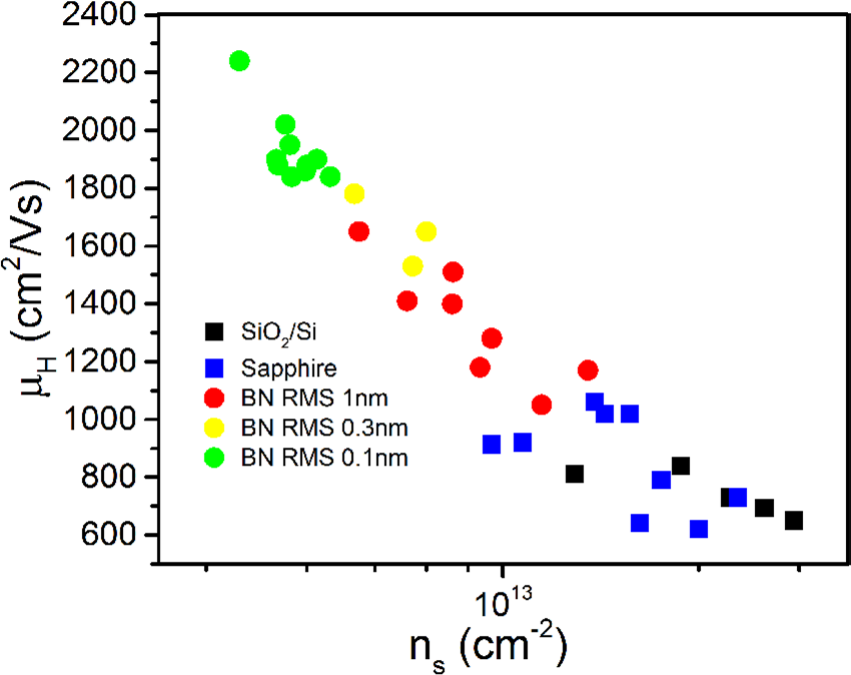
Room temperature Hall analysis of graphene on various dielectric substrates. Measured Hall effect mobility vs. sheet carrier density.

To probe the effects of the dielectric substrate on graphene transport, room temperature Hall measurements were performed on more than 35 1 cm × 1 cm graphene samples on various substrates. Regardless of substrate material, all samples were found to be p-type. Figure 3a shows Hall mobility (µ_H_) as a function of sheet carrier density (n_s_) for each sample. As a comparison, graphene transferred to c-plane sapphire and SiO_2_/Si substrates was also investigated. From this data a clear separation into groups based on the substrate can be observed, illustrating the impact the underlying substrate has on transport. Graphene films on sapphire and SiO_2_/Si have the lowest µ_H_ and highest n_s_. By switching to even a very rough (>1 nm) BN/sapphire substrate a noticeable reduction in n_s_ and increase in µ_H_ is achieved. This trend of improving graphene transport continues as the BN roughness is reduced. A 2x increase in µ_H_ and one order of magnitude decrease in n_s_ was achieved on the smoothest BN/sapphire substrates. The substrate is known to effect n_s_ and µ_H_ in a number of ways causing doping, phonon scattering and/or corrugation of the graphene layer. At the surface of SiO_2_ and sapphire hydroxyl groups can form^31,32^, which will adsorb water and p-type dope the graphene^33,34^. Switching to the inert vdW surface of BN will eliminate this substrate doping effect assuming a near perfect surface and interface. For graphene supported by MOCVD BN/sapphire we expect a contribution to n_s_ from surface impurities and defects, which may form hydroxyl groups similar to sapphire and SiO_2_. As such, it is not surprising to see a higher n_s_ in graphene supported by rough BN. If we compare the morphologies and layer orientation (Fig. 1) for the different BN/sapphire substrates, we can visualize how improving surface roughness and morphology may reduce surface defects contributing to n_s_. The corresponding increase in mobility with decreasing roughness can be attributed to multiple factors. Most obviously from Fig. 3, mobility is inversely affected by n_s_, which as previously reported for CVD graphene on BN may been due to the reduction in charged impurity Coulomb scattering^35,36^. The additional effects of substrate roughness and phonons may also contribute, as will be explored below.

For substrate supported graphene, scattering due to the surface impurities^37^, roughness^38^, and surface optical (SO) phonons^3^ from the substrate as well as graphene longitudinal acoustic (LA) phonon^2^ and short range scattering^39^ will all effect mobility. The mobility limited by Coulomb scattering from impurities, short range scattering from defects and roughness are all temperature independent mechanisms that will be defined here as µ_0_. The temperature dependent mechanisms for the graphene LA phonon (µ_AL_) and substrate SO phonon (µ_SO_) can be expressed as:

1 $${\mu }_{LA}=\frac{eh{\rho }_{s}{v}_{s}^{2}{v}_{f}^{2}}{n{\pi }^{3}{D}_{A}^{2}{k}_{b}T}$$

2 $${\mu }_{SO}=\frac{C}{ne}({e}^{({E}_{0}/{k}_{b}T)}-1)$$

where *ρ*_*s*_ *is* the graphene 2D mass density, v_f_ is the fermi velocity, v_s_ is velocity of sound, D_A_ is the acoustic deformation potential, E_0_ is the substrate phonon energy, and C is the coupling strength. Using Matthiessen’s rule, the total limited mobility is expressed as 1/µ_tot_ = 1/µ_0_ + 1/µ_LA_ + 1/µ_SO_. Accordingly, we observe an inverse mobility dependence on n_s_ (Fig. 3). At high temperatures the substrate SO phonons will have a strong effect on the temperature dependence of µ_tot_. Comparison of the lowest E_0_ values, taken from literature, for BN (169 meV)^8^ and sapphire (55 meV)^40^ shows that switching to a BN/sapphire substrate can positively impact µ_H_ at room temperature.

**Figure 4:**
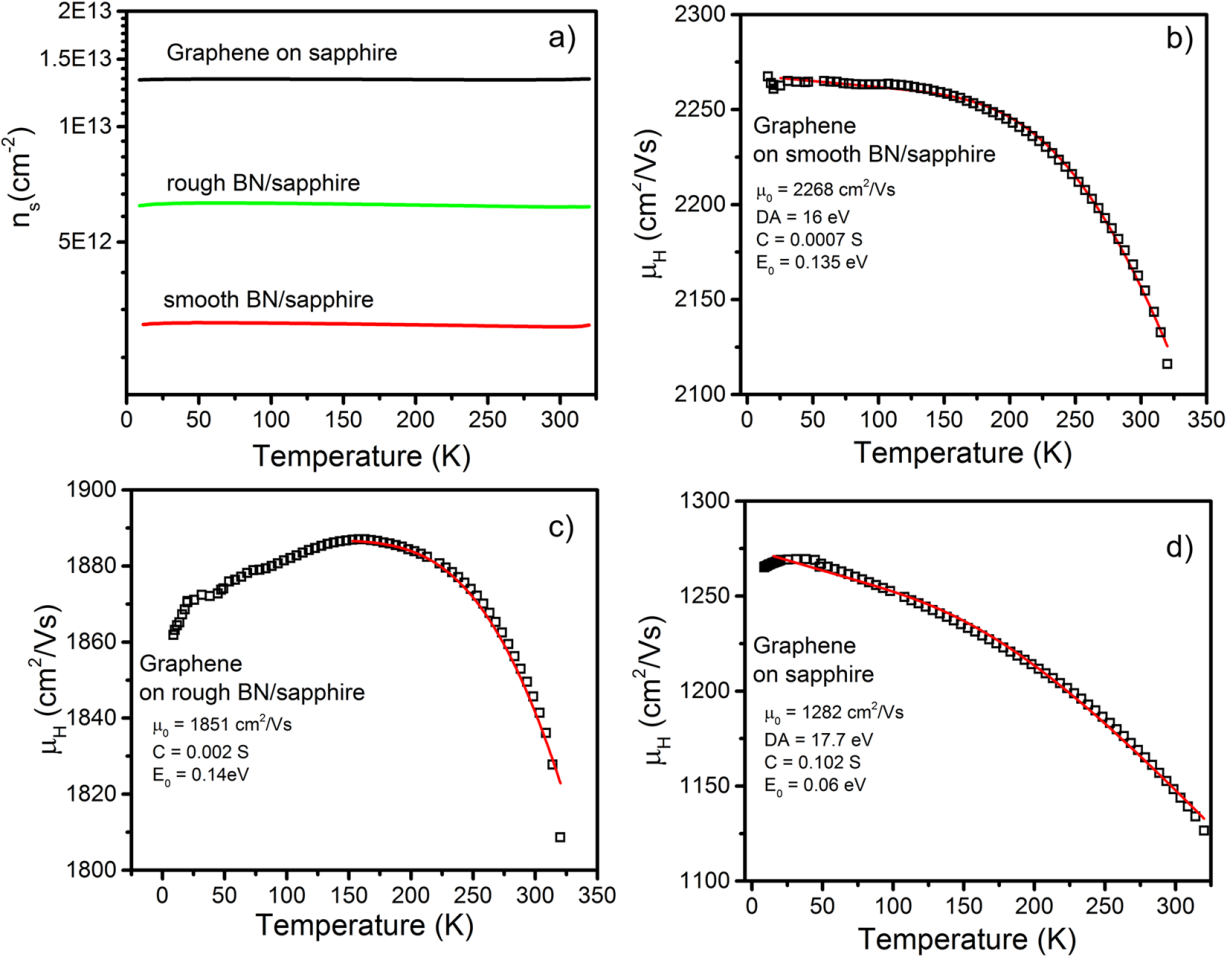
Analysis of graphene transport on various dielectric substrates. (**a**) Temperature dependence of graphene sheet carrier density. Temperature dependent mobility of graphene on (**b**) sapphire, (**c**) rough BN/sapphire, and (**c**) smooth BN/sapphire. Red lines are fits to µ_tot_ = (1/µ_0_ + 1/µ_LA_ + 1/µ_SO_)^−1^. Fitting parameters µ_0_, DA, C, and E_0_ are given for each sample.

Further studying the effects of BN on graphene transport, temperature dependent Hall measurements were performed on graphene samples on sapphire, rough BN/sapphire and smooth BN/sapphire substrates. From all samples, we find n_s_ to be independent of temperature over the range of 10 to 320 K (Fig. 4a) indicative of the 2D nature of graphene. The substrate dependence of n_s_ further illustrates the strong contribution of surface charge and impurities to graphene. Comparison of n_s_ for graphene on rough vs. smooth BN suggests a higher concentration of surface charges on the rough BN with more random layer morphology as observed in room temperature measurements.

Temperature dependent mobility for graphene on sapphire, rough BN, and smooth BN are shown in Fig. 4b–d. From initial inspection, a clear difference between samples can be observed. The graphene films on smooth BN and sapphire substrates show the typical trend of increasing mobility with decreasing temperature over the full range of measurement, while on the rough BN/sapphire substrate a decrease in mobility with temperature is observed at temperatures below ~150 K. Although both the graphene on smooth BN/sapphire and sapphire have the same inverse temperature dependence, we clearly see that on sapphire mobility continues to increase up to ~20 K, while on smooth BN mobility increases until only ~125 K. As described in Eqn. (2) this is due to the difference in E_0_ for BN and sapphire. Fitting of the measured Hall mobility to µ_tot_ = (1/µ_0_ + 1/µ_LA_ + 1/µ_SO_)^−1^ shows good agreement with graphene on both smooth BN and sapphire. Only µ_0_, D_A_, C, and E_0_ were obtained from fitting given in Fig. 4b–d. All other constants were taken from literature (Supplementary Table S1). The values for D_A_ were determined to be 17.7 and 16 eV for sapphire and smooth BN substrates in good agreement with theoretical and experimental values^2^. The experimentally determined values for E_0_ are 135 meV for the smooth BN/sapphire and 60 meV sapphire. On a rough BN/sapphire substrate, graphene mobility is not well described by Eqns. (1) and (2), which both have an inverse temperature dependence. Instead, a positive temperature dependence below 150 K is observed, more indicative of a 3D scattering mechanism like ionized impurity scattering. Positive temperature dependence of mobility has been observed in highly defective irradiated^41^ or fluorinated^42^ graphene. This thermally activated transport in highly defective graphene has been attributed to localization effects. As discussed above, the high substrate roughness is expected to produce local defects in the graphene, which may cause a similar effect, but more work is needed to further elucidate this phenomenon. Similarly, transport has also been observed in graphene on SiC due to parallel conduction in the 2D graphene and 3D SiC substrate^43^. We do not expect to see any contributions to electrical measurement from the BN/sapphire substrate due to the highly insulating nature of both BN and sapphire. Attempts to measure sheet resistivity in BN/sapphire substrates give values greater than 10^10^ Ω (system limit). Since at higher temperatures mobility is limited by substrate induced SO phonon scattering, we fit the graphene on rough BN mobility to µ_tot_ = (1/µ_0_ + 1/µ_SO_)^−1^ for temperatures greater than 150 K. This fit gives us an estimate of E_0_ (140 meV) for these BN/sapphire substrates, which is in good agreement with the smooth BN/sapphire. Using this modified version of µ_tot_ we also obtain a value of 1851 cm^2^/Vs for µ_0,_ which is between those for the smooth BN and sapphire. Although, this term contains the contribution of roughness to µ_tot_ we cannot explicitly determine its contribution considering Coulomb and short range scattering are also temperature independent. If we qualitatively compare µ_0_ from the graphene on smooth BN and sapphire samples, which have similar roughness (<0.1 nm), we find the effects of impurity and carrier density (Coulomb and short range) to be significant suggesting a stronger contribution to µ_tot_ than roughness.

**Figure 5:**
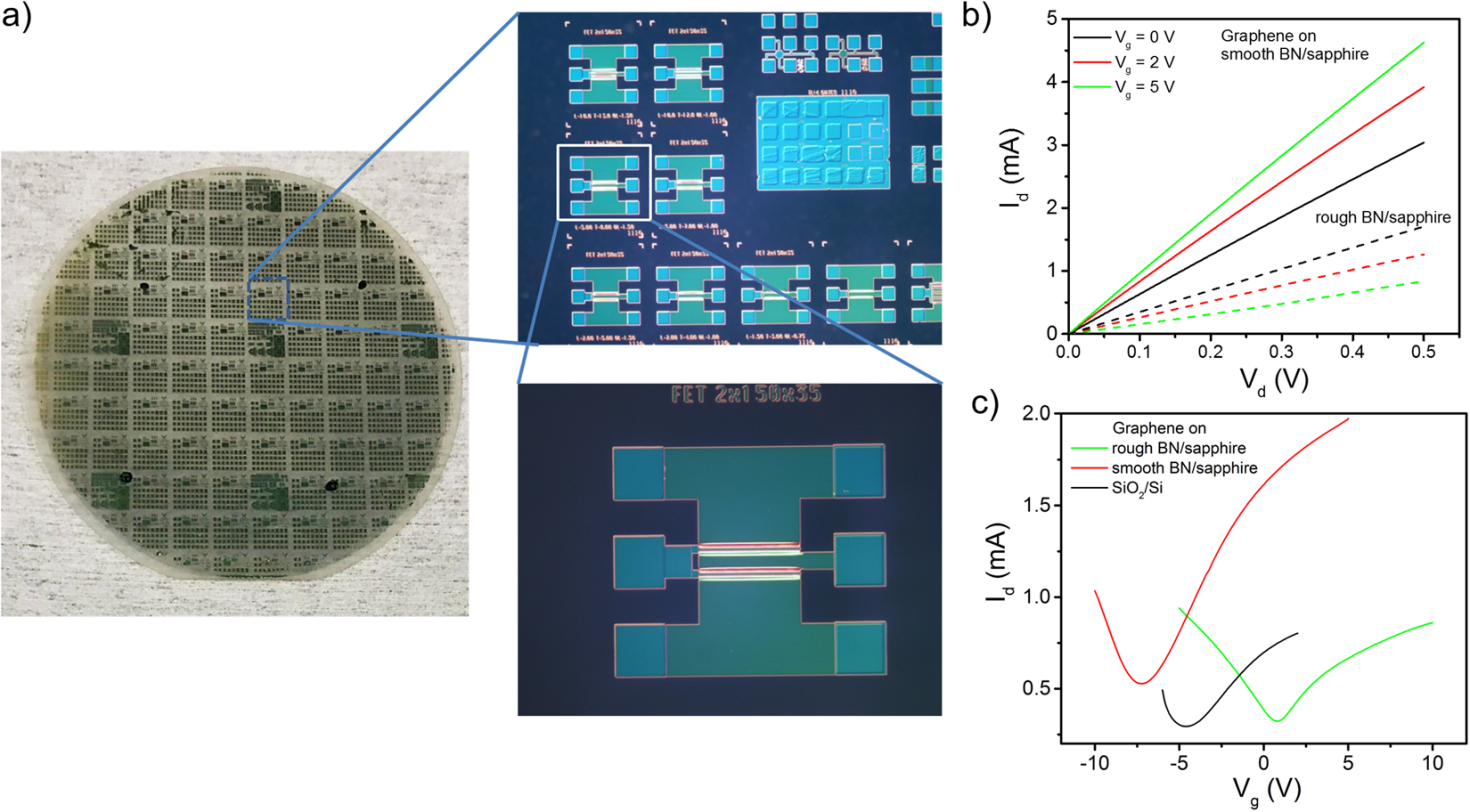
Graphene FET fabrication and characterization. (**a**) Optical images of processed 2” wafer and zoom in on devices. (**b**) Output and (**c**) transfer curves from representative devices with a 10 µm channel length on smooth, rough BN/sapphire and SiO_2_/Si.

Further quantifying MOCVD grown BN as a substrate for graphene devices, top-gated FETs were fabricated on a 1” graphene film transferred to a 2” BN/sapphire substrate (Fig. 5a). The mask design included a large array of device dimensions; however, yield on devices with channel lengths <2 µm was very low. As a result, this work focused on devices with channel lengths 2 to 10 µm. As a comparison, graphene on SiO_2_/Si FETs were also fabricated and tested. Typical I_d_-V_d_ measurements showing gate modulation in devices on smooth and rough BN/sapphire are shown in Fig. 5b. Gate modulation in devices on smooth BN/sapphire shows a positive dependence on gate voltage, indicating electrons as the majority carrier. In devices on rough BN/sapphire the modulation has a negative dependence on gate voltage indicating the majority carriers are holes. The observed n-type nature of graphene (g)FETs on the smooth BN/sapphire is contrary to the p-type nature observed in the Hall measurements. Since Al_2_O_3_ has been shown to dope graphene and can significantly increase electron concentration, it is expected that the deposition of the Al_2_O_3_ top gate is related to this difference in majority carrier type^44^. The observed switch (from p to n) in majority carrier type in devices on smooth BN/graphene, but not rough BN/sapphire, is due to the lower background carrier density.

The transfer characteristics of gFET devices on rough BN/sapphire, smooth BN/sapphire and SiO_2_/Si were also studied. The Dirac point voltage is clearly different for these three dielectric substrates, Fig. 5c. Controlled doping is not easy in graphene and the Dirac point of gFETs cannot readily be adjusted by tuning the doping density of the graphene channel. However, as shown here the material in contact with the graphene channel can dictate the type of charged carriers. The Dirac point of a graphene device can be adjusted by selecting a gate metal with suitable work function and/or having a large gate capacitance to ensure a strong response between the gate electrode and graphene channel^45^. In our case, the bottom BN layer in the devices presented here also changes the Dirac point. Figure 5c shows the transfer characteristics for the three sets of devices. The Dirac point for devices on smooth BN/sapphire is ~ −7 V, while for devices on rough BN/sapphire and SiO/Si it is about 1 and −4.5 V, respectively. Devices show the typical ambipolar gFET behavior as manifested by the “V” shape of the transfer curve. A slight shift in the Dirac point with the drain voltage was observed due to hysteresis in the channel. This is likely from impurities and defects at the graphene-substrate and graphene-gate interfaces^46^.

Next, field effect mobility values for devices with different dimensions were extracted to determine the uniformity of our process. One common method is to use transfer curves to extract mobility using the following equation

3 $$\mu ={{\rm{g}}}_{{\rm{m}}}.{\rm{L}}/{{\rm{V}}}_{{\rm{d}}}.{\rm{W}}.{{\rm{C}}}_{{\rm{ox}}}$$

where g_m_ is transconductance, defined as g_m_ = dI_d_/dV_g_, C_ox_ is the gate oxide capacitance (C_ox_ = κε_0_/t_ox_), W is channel width, and L channel length. Using this method, we found mobility depended strongly on the channel length (Supplementary Fig. S6). This significant variation in mobility is largely due to effects of contact resistance, which can obscure the true field effect mobility in transistor devices. The so-called Y-function method^47^ has been used has an effective way to limit effects of contact resistance in extracting field effect mobility in low dimensional FETs^48,49^. This method relies on combining drain current and transconductance transfer characteristics considering the first order mobility attenuation coefficient and contact resistance. Assuming contact resistance does not depend on V_g_ Y-function is defined as:

4 $$Y=\frac{{I}_{d}}{\sqrt{{g}_{m}}}=\sqrt{\frac{\mu {C}_{ox}{V}_{d}W}{L}}{V}_{G}.$$

**Figure 6:**
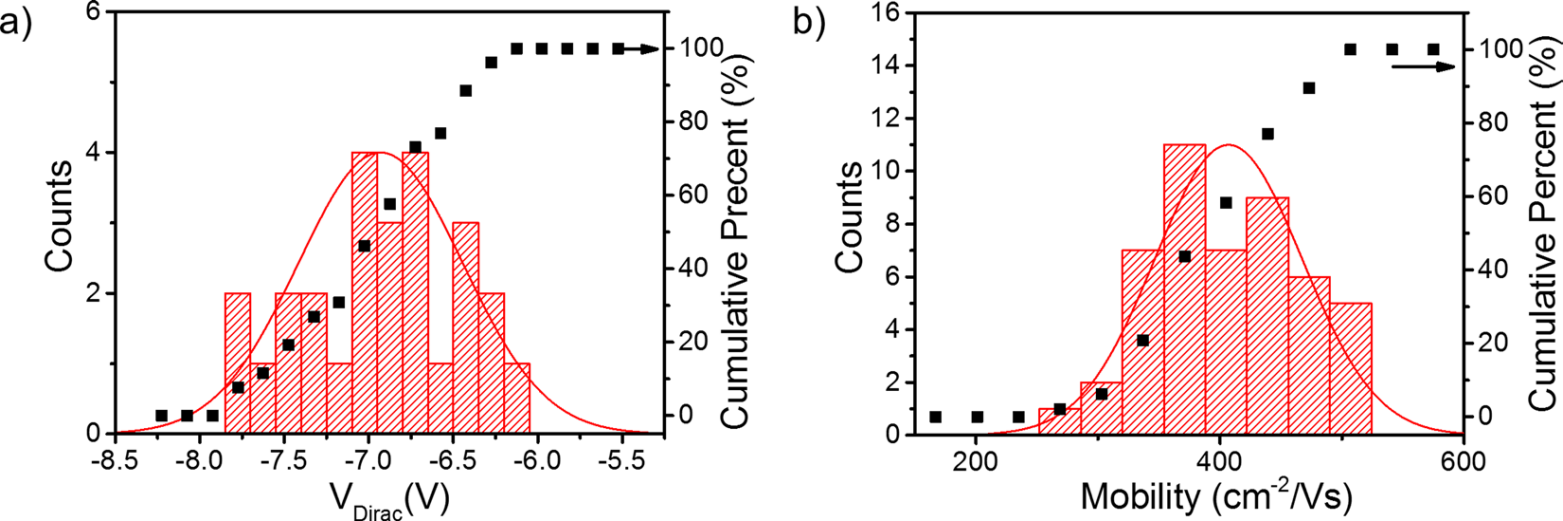
Histogram and cumulative percent plots of extracted (**a**) Dirac voltage and mobility from 50 devices with channel length between 2 and 10 µm on smooth BN/sapphire.

Plotting Y against V_g_, mobility can be extracted from the slope of the linear portion of the curve. The mobility values extracted from this method are more consistent than those calculated using the transconductance method. We characterized more than 50 devices with channel lengths from 2 to 10 µm across the wafer. Figure 6 shows the distribution of the Dirac voltage and extracted mobility across the graphene FET array on a smooth BN/sapphire wafer. A mean V_Dirac_ of −7 with a standard deviation of only 1 V is quite narrowly distributed indicating relatively good carrier uniformity. A mean extracted µ of 409 with a standard deviation of 60 cm^2^/Vs was also measured. For comparison FETs on rough BN/sapphire and SiO_2_/Si have a typical mobility of 260 and 96 cm^2^/Vs, respectively. These values are considerably lower than those measured using the Hall effect, which is believed to be caused by impurities and graphene degradation from the top gate fabrication process. One way to reduce process degradation and doping from Al_2_O_3_ deposition would be to cap the graphene surface with a thin BN layer prior to processing. The narrow distribution of these typical device metrics highlights the uniformity of the BN/sapphire substrate and its usefulness as a weakly interacting substrate for graphene devices.

## Conclusion

We have presented results from a detailed study on MOCVD BN on sapphire as a weakly interacting dielectric substrate for graphene. Wafer scale BN can be grown on sapphire with low roughness and excellent uniformity. Using Raman, Hall effect, and FET device characterization we studied the impact the dielectric substrate has on CVD transferred graphene. A greater than 10x decrease in graphene carrier density and 2x increase in mobility is achieved by using a BN/sapphire substrate as compared to SiO_2_/Si or sapphire. Scattering due to increased n_s_ and SO phonons from the substrate were found to have the most significant impact on mobility at room temperature. Substrate roughness was also found to be correlated with graphene doping. Large area (1” square) graphene FET arrays were fabricated on 2” BN/sapphire and investigated showing good uniformity across the array. This collective work demonstrates significant performance improvements in graphene using scalable methods for producing BN/sapphire substrates.

## Methods

Boron Nitride thin films used in this study were grown via a metalorganic chemical vapor deposition (MOCVD) technique. The substrates used for BN growth were c-plane sapphire which allows for uniform, few layer BN to be grown. In a cold-walled vertical MOCVD reactor, the NH_3_ and TEB precursor gases used for N and B, respectively, are injected such that they are not heated nor do they interact with one another until they reach the surface of the substrate. The reactor was kept at a pressure of 20 Torr and the substrate was heated to 1000 °C during the growth. The ratio of NH_3_ to TEB was set between 2250 to 950 to achieve the desired morphology and quality of BN films.

Graphene was grown on a copper substrate by CVD using methane as the carbon source. Films were subsequently transferred to dielectric substrates using a typical wet transfer with PMMA, similar to the process described in ref.^50^. After transfer, graphene films were annealed in forming gas (5% H_2_: 95% Ar) for 1 hr at 300 °C to remove any additional residue from the transfer process.

Transferred graphene and BN MOCVD layers were characterized using a suite of techniques. The BN thickness was measured by X-ray reflectance (XRR) using a PANalytical Empyrean X-ray diffractometer at a grazing incidence angle. Cross section TEM samples were prepared using the *in-situ* FIB lift out technique on an FEI Dual Beam FIB/SEM and imaged with a FEI Tecnai TF-20 FEG/TEM operated at 200 kV in high-resolution (HR) TEM mode. The surface morphologies of the graphene and BN films were analyzed by AFM using a Bruker Dimension Icon in tapping mode. Raman measurements were performed using a Renishaw inVia system under a backscattering geometry with scattered radiation collected along the sapphire [0001] direction. A 4 mW 488 nm excitation source, 20 µm slits, and a 3000 l/mm grating was used for each measurement. The transport properties were characterized by Hall-effect measurements. For room temperature measurements, an Accent 5500 system was used under ambient conditions. For temperature dependent measurements from 10 to 320 K, a Lakeshore 7507 system was used. Prior to measurement the sample was loaded into the cryostat, heated to 350 K under a He atmosphere and held overnight. Measurement sweeps were taken during cooling to 10 K and heating to insure a consistent measurement. If samples were not heated to 350 K, the measurements were found to be quite different on heating and cooling.

Top-gated graphene FETs were fabricated on transferred graphene on BN/sapphire and SiO_2_/Si substrates. Graphene mesas were defined using O_2_-plasma etch followed by source/drain metal contacts (Ti/Pt/Au) using a first lift-off process. A 20-nm thick Al_2_O_3_ was used as gate dielectric. Finally, 100-nm thick gate metal (Ti/Au) contacts were defined using a second lift-off process. Gate dimensions were chosen such that electron beam lithography process is not required for the FET fabrication process. An Agilent 4156 C precision semiconductor parametric analyzer was used for all the electrical characterization of the FETs.

## Electronic supplementary material

Supplementary information
