# Supplementary material for: Wafer scale BN on sapphire substrates for improved graphene transport
Source: Sci Rep. 2018 Jun 11;8:8842. doi: 10.1038/s41598-018-27237-z (PMC5996022; doi:10.1038/s41598-018-27237-z)
Supplement: Supplementary file 1 — Supplementary information [file 41598_2018_27237_MOESM1_ESM.pdf]

Supplementary Information:

Wafer scale BN on sapphire substrates for improved graphene transport

Shivashankar Vangala,[1] Gene Siegel,[1,3] Timothy Prusnick,[1,2] Michael Snure[1,*]

1) Air Force Research Laboratory, Sensors Directorate, Wright Patterson AFB, 45433, USA

2) Wyle Laboratories, Beavercreek, OH 45433 USA

* E-mail: michael.snure.1@us.af.mil, Tel: +1-973-528-8929

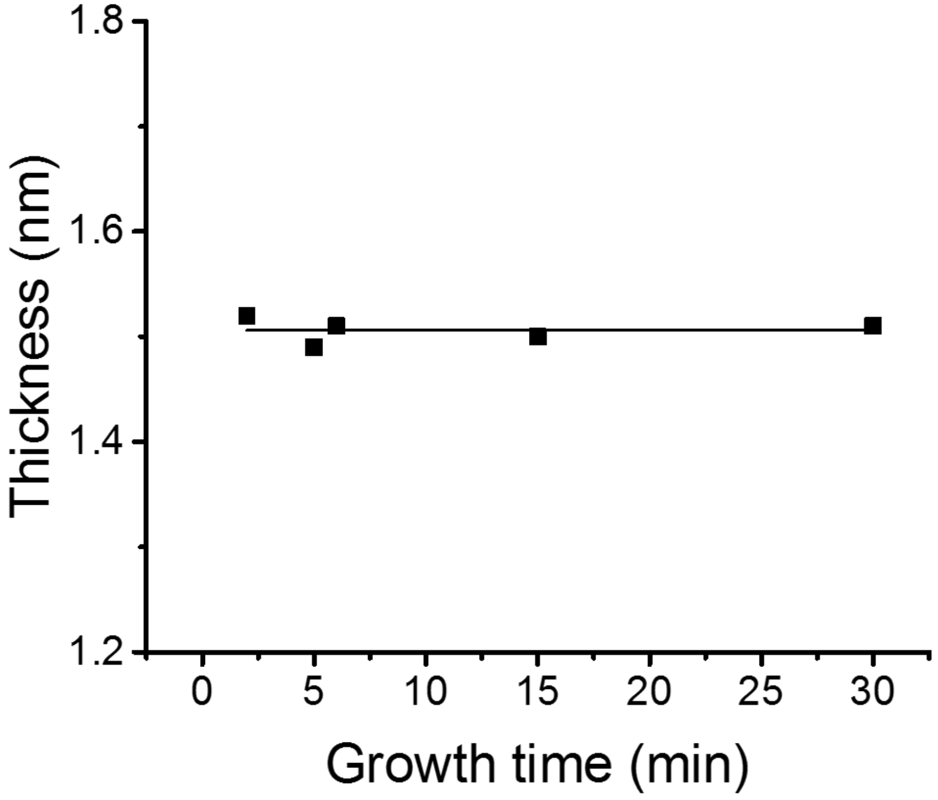

Figure S1: BN thickness as a function of growth time for BN grown on sapphire at 1000 °C, 20 Torr, and V/III =2250.

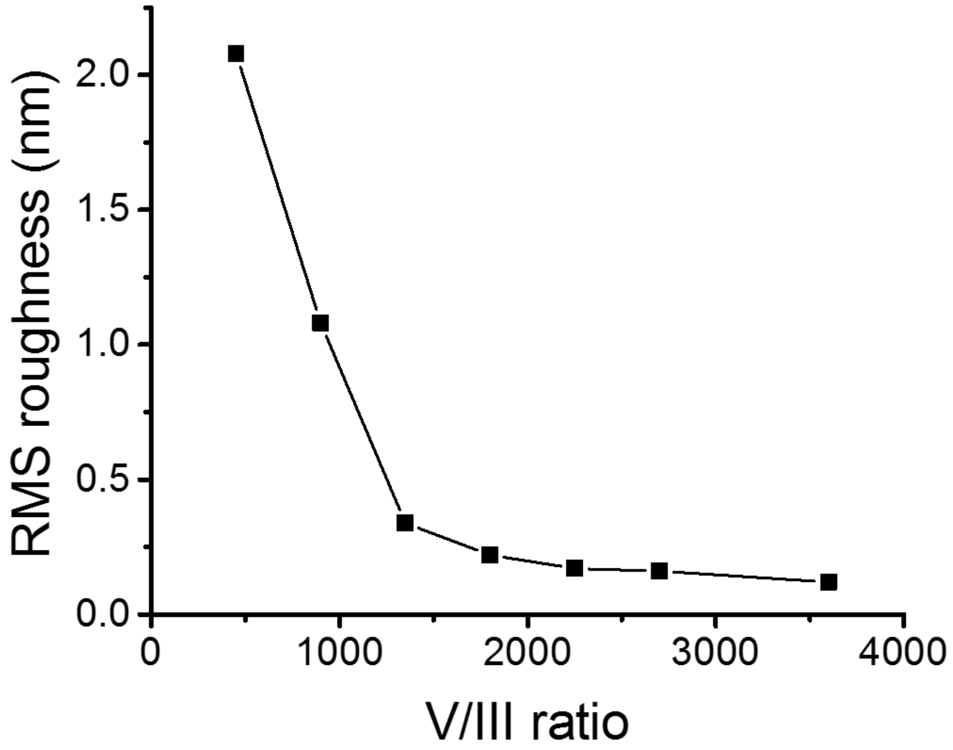

Figure S2: BN on sapphire roughness determined by AFM as a function of V/III ratio.

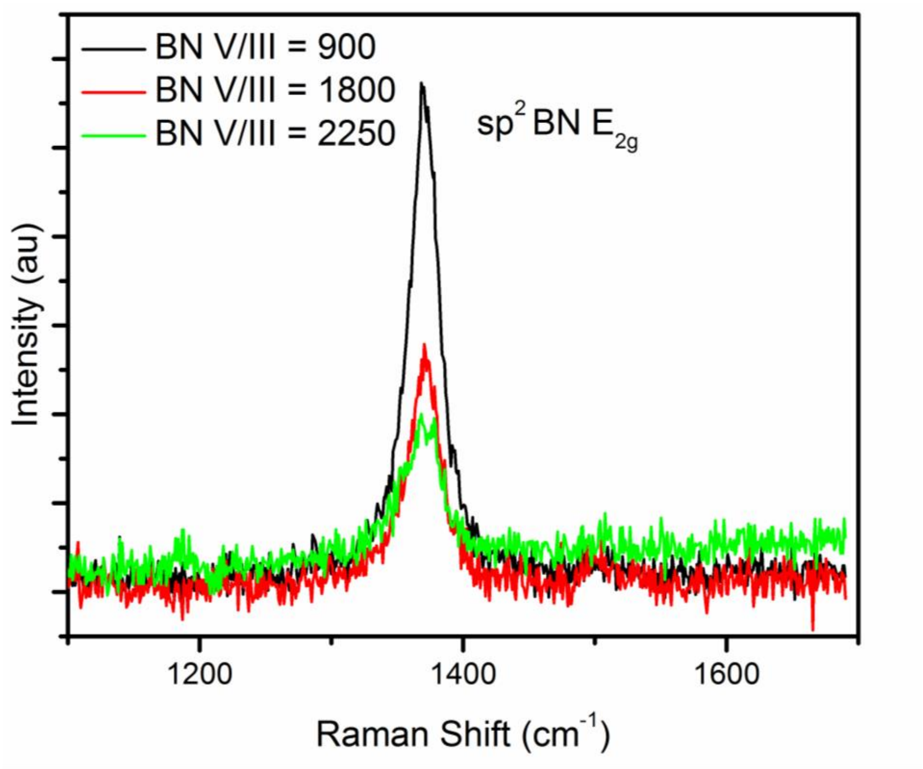

Figure S3: Raman spectroscopy from MOCVD BN on sapphire grown at different V/III ratios.

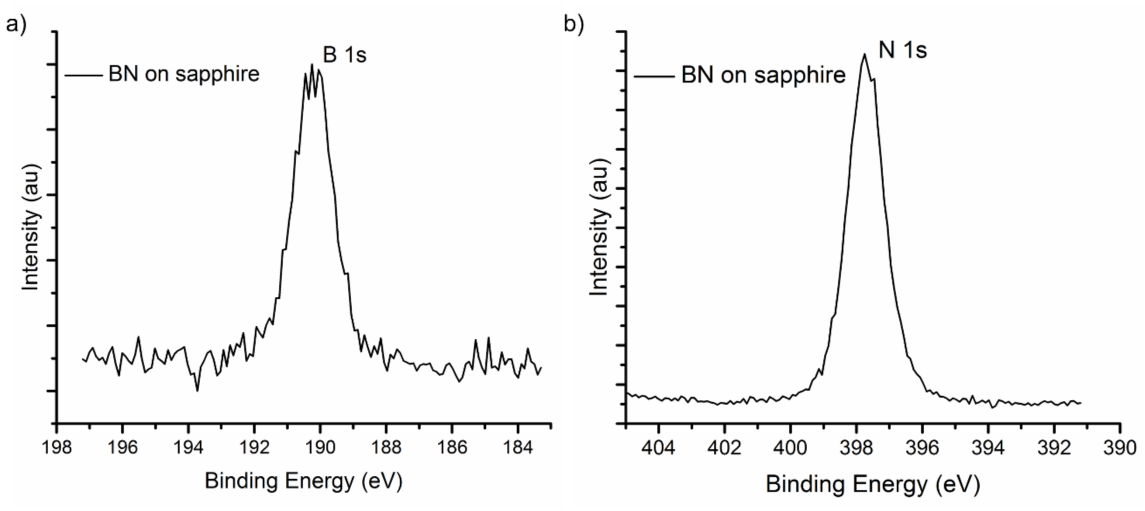

Figure S4: X-ray photoelectron spectroscopy from BN on sapphire grown at a V/III ratio of 2250.

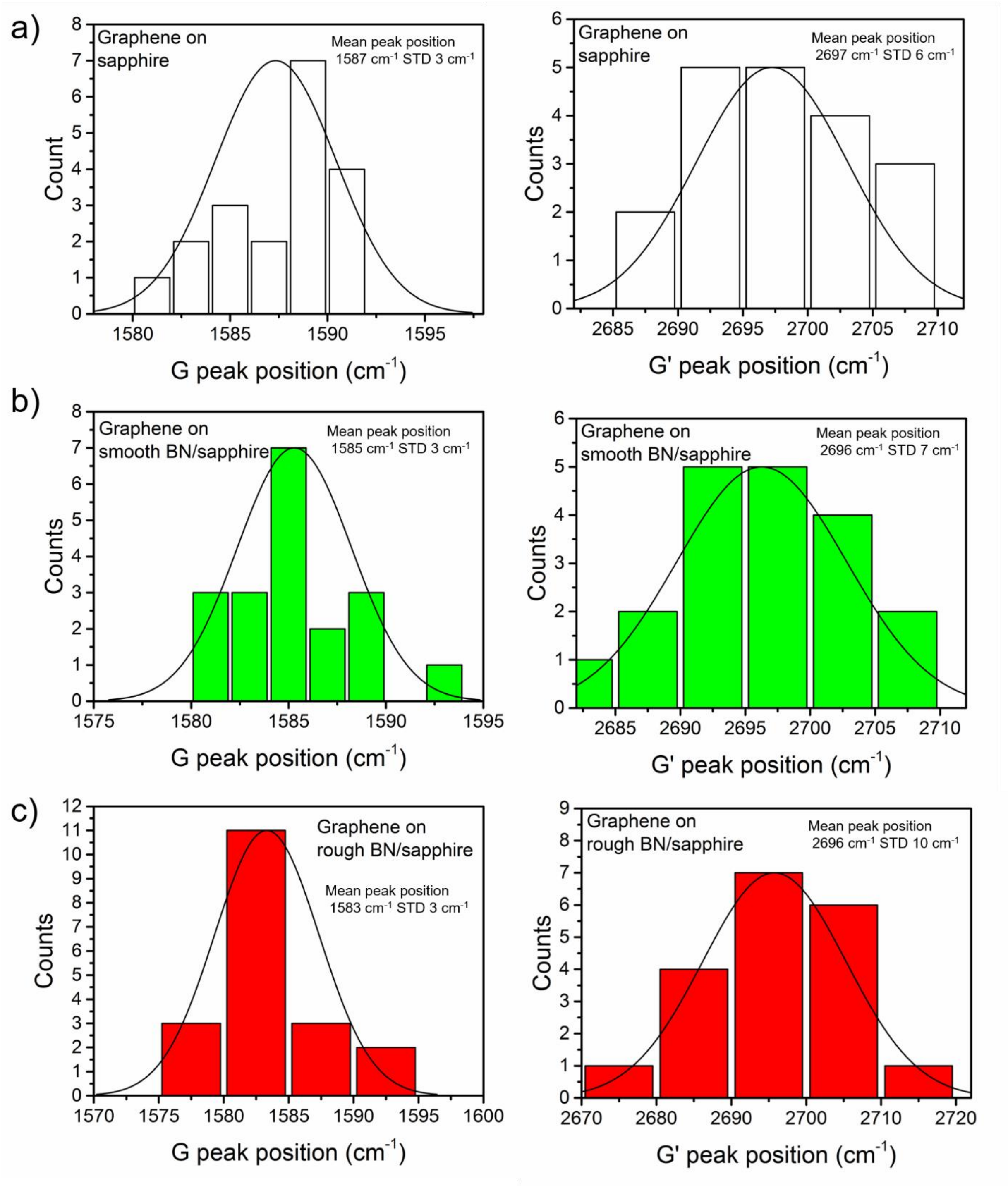

Figure S5: Raman analysis of graphene on various dielectric substrates. Measured G and G’ peak position distribution from graphene films on a) 6 sapphire, b) 11 smooth BN/sapphire, and c) 9 rough BN/sapphire substrates. The mean peak position and standard deviation (STD) is given for each.

Table S1: Graphene constants taken from ref. [2]

ρ_s - graphene 2D mass density	7.6 x 10^{-7} kg m^{-2}
v_f -fermi velocity	10^6 m/s
v_s - velocity of sound	2.1 x 10^4 m/s

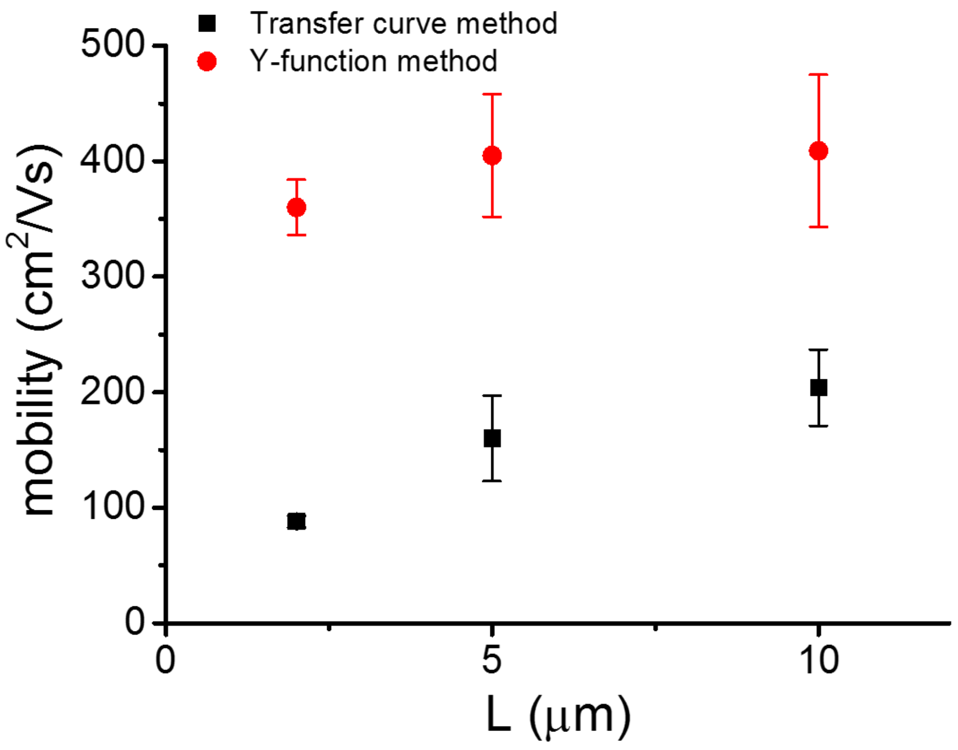

Figure S6: FET mobility as a function of channel length for devices on smooth BN/sapphire. Mobility extracted using transfer curve and Y-function methods.
